# An improved and uptaded 20-years air pollution exposure dataset for Germany 2003-2022 (APExpose_DE v6)

**DOI:** 10.1016/j.dib.2025.111833

**Published:** 2025-06-24

**Authors:** Alexandre Caseiro, Erika von Schneidemesser

**Affiliations:** RIFS Research Institute for Sustainability at GFZ, Germany

**Keywords:** Air quality, Exposure, Health, Germany

## Abstract

The link between exposure to polluted air and the outcome of diseases (e.g., cardio-vascular diseases, COVID-19) has been established. Nevertheless, research on the quantification of the relationship is still relevant today. Quantifying the link between the ambient atmospheric concentrations of pollutants and the outcome of diseases requires knowledge on the levels of the pollutants through time at various scales. In the present work, an improved and updated version of the APExpose_DE dataset is described. The dataset provides air pollution metrics at the yearly time resolution and at the spatial resolution of the NUTS-3 level, corresponding to the *Landkeis/Kreisfreie Stadt* in Germany. The dataset evolved from its initial form by expanding the years covered and by refining the gap-filling methodology. The dataset can serve as input to, e.g., observational studies.

Specifications Table

**Table:** 

Subject	Atmospheric Science
Specific subject area	The present dataset provides air pollutant concentation estimates in Germany at the spatial level of the NUTS-3 and at the temporal resolution of one year for the years 2003-2022, relevant for exposure studies.
Type of data	Table Processed
Data collection	Ambient concentrations of atmospheric pollutants measured in Germany in 2003-2022 and reported to the European Environmental Agency were retrieved from the Air Quality Download Service (formerly known as Airbase, under a different architecture). The data was processed and completed with reanalysis data from the Copernicus Atmosphere Monitoring Service, after scaling, in order to estimate the monthly concentrations to which people were subjected to for each county in Germany.
Data source location	The data were collected from the European Environmental Agency through its Air Quality Download Service (https://eeadmz1-downloads-webapp.azurewebsites.net/) and from the Copernicus Atmosphere Monitoring Service through its Atmosphere Data store (https://ads.atmosphere.copernicus.eu/datasets/cams-global-reanalysis-eac4?tab=download).
Data accessibility	Repository name: Zenodo Data identification number: 10.5281/zenodo.14873210 Direct URL to data: https://zenodo.org/records/14873210
Related research article	10.1186/s12940-024-01149-0

## 1 Value of the Data

• The present data provides long-term air pollution exposure values at a spatial resolution (the NUTS-3 units) which corresponds to health records, or to which health records can easily be aggregated. The temporal resolution of the data is yearly. These three characteristics (temporal and spatial resolutions as well as temporal depth) make the dataset especially useful as input for observational studies, or as a validation dataset for air quality modelling studies.
• Besides its temporal and spatial resolutions as well as its temporal depth, the ASCII format of the provided dataset enables a simple access and workup (e.g., it can be easily rasterized or vectorized).
• The characteristics of the dataset makes it useful for investigating policy; the evolution of exposure to atmospheric pollutants can be evaluated in concert with the evolution of the policy.

## 2 Background

Despite the link between air pollution and adverse health outcomes being well established, high-quality datasets are needed to improve its quantification [1]. The present dataset is an updated and improved version of the dataset described in Caseiro and von Schneidemesser [2]. The first version of the dataset was aimed at supporting observational studies such as the one developed by Koch et al. [3]. The years covered were gradually expanded (through versions 2, 3 and the current one, 6) from 2010–2019 to 2003–2022, now covering 20 years. The downscaling of the CAMS (Copernicus Atmosphere Monitoring Service) reanalysis data was refined through the versions, from a simple linear regression between reanalysis and monitoring-based values (versions 1 and 2), to a multilinear regression further including population, area, and population density of the NUTS-3 units (version 3), to finally including the area type of each NUTS-3 unit as a factor (version 4 onwards). Each NUTS-3 unit can be classified into one of four classes, as defined by the Federal Institute for Research on Building, Urban Affairs and Spatial Development (*Bundesinstitut für Bau- Stadt- und Raumforschung*), based on estimations of the population actually present within the unit throughout a typical day: very central, central, peripheral or very peripheral.

## 3 Data Description

The provided dataset comprises the following files:

• APExpose_DE__2003-2022__changelog.md: a short description of the updates throughout the versions.
• APExpose_DE__2003-2022.README: a short description of the dataset in APExpose_DE__2003-2022__nogeo.csv (description of the fields, records and metadata).
• APExpose_DE__2003-2022__nogeo.csv: the air pollution concentration for exposure estimates data itself. Each record (each line in the file) corresponds to a NUTS-3 unit (identified by its name and its code), and a scenario, for a given year. There are 402 NUTS-3 units in Germany and 3 scenarios were developed, the total number of records in the dataset is 1206 per year, or 24120 for the entire study period. Each record includes a numeric value for each metric considered.
• APExpose_DE__2003-2022__Ratified.csv: describes the level of QA/QC for the original monitoring data.
• APExpose_DE__2003-2022__StationTypes.csv: describes the station(s) used for each data point, in the case of monitoring data, or indicates the use of reanalysis data.

The variables present in the dataset (file APExpose_DE__2003-2022__nogeo.csv) are:

• NO_2_ annual mean concentration (micrograms per cubic meter)
• number of hours of the year which have an NO_2_ concentration over 200 μg per cubic meter
• NO annual mean concentration (micrograms per cubic meter)
• PM10 annual mean concentration (micrograms per cubic meter)
• number of days of the year which have a daily average PM10 concentration over 50 μg per cubic meter
• PM2.5 annual mean concentration (micrograms per cubic meter)
• O_3_ annual mean concentration (micrograms per cubic meter)
• number of days of the year which have a daily average O_3_ concentration over 120 μg per cubic meter
• annual mean of the daily O_3_ maximum concentration (micrograms per cubic meter)
• maximum daily 1 h average O_3_ concentration over the entire year (micrograms per cubic meter)
• maximum daily 8 h average O_3_ concentration over the entire year (micrograms per cubic meter)

## 4 Experimental Design, Materials and Methods

In all versions of APExpose_DE so far, the NUTS-3 units for Germany reflect the status until November 1st, 2016 (402 units), when the two Landkreise Göttingen and Osterode am Harz merged. Two sources were used for the production: (1) measurements from the Air Quality Download Service (formerly Airbase), managed by the European Environmental Agency, and (2) the CAMS global reanalysis EAC4.

### 4.1 Airbase

The monitoring data, accessed between 2020 and 2023 via the EEA's Air Quality Download Service (formerly, under a different architecture, known as Airbase), was processed in the same way as described by Caseiro and von Schneidemesser [2] with the exception of expanding to the years 2003-2022. The data sources were as follows:

• 2003–2012: the Airbase-v8 dataset
• 2012–2021: the E1a dataset (ratified)
• 2022: the E2a dataset (unratified)

### 4.2 CAMS

The yearly averaged CAMS reanalysis data was processed (clipped and vectorized) to be used for gap-filling. In the first version the processed reanalysis data was scaled via a simple linear regression using the NUTS-3 unitwise measurements-based data as dependent variable and the processed reanalysis-based data as independent variable. In the current version multilinear regressions were constructed adding the NUTS-3 population and/or its area and/or its population density and/or its structural settlement type, as defined by the Federal Institute for Research on Building, Urban Affairs and Spatial Development (*Bundesinstitut für Bau- Stadt- und Raumforschung*, https://www.bbsr.bund.de/BBSR/DE/forschung/raumbeobachtung/Raumabgrenzungen/deutschland/kreise/Raumtypen2010_krs/Raumtypen2010_Kreise.html), as independent variables. The best performing model was selected using the Akaike Information Criterion and used to estimate concentrations for the NUTS-3 units which did not have measurements.

CAMS-based data for the metrics with a time resolution shorter than one day were produced as described in Caseiro and von Schneidemesser [2].

### 4.3 APExpose_DE

**Fig. 1:**
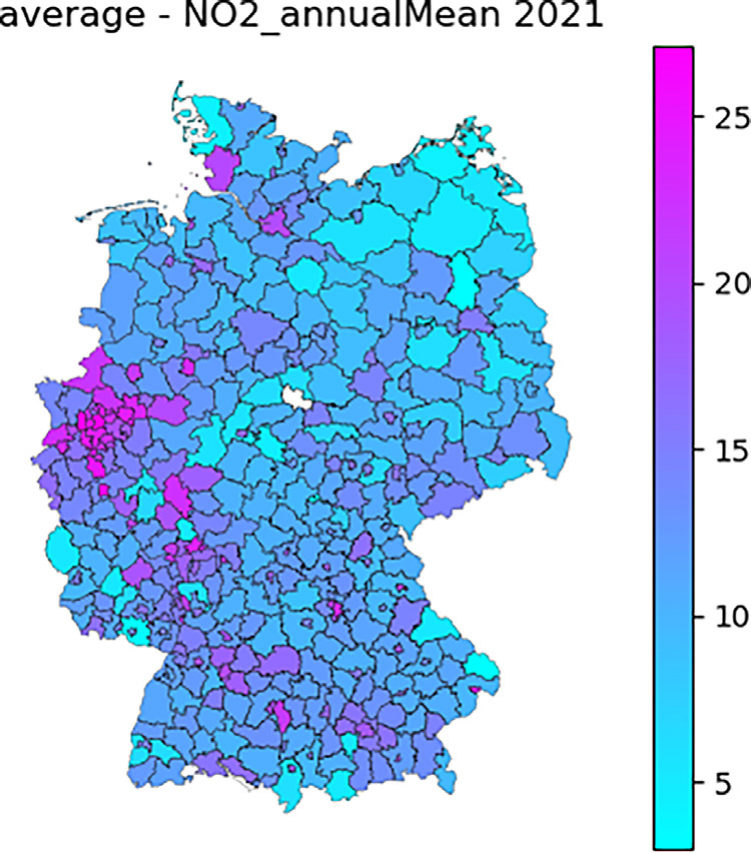
annual average NO_2_ concentration (µg.m^-3^) for 2021.

As a final step, the Airbase- and CAMS-derived data are combined to produce the version 6 of the APExpose_DE dataset. An example is given in Fig. 1: the NO_2_ annual mean for 2021 (average scenario).

## Limitations

The main drawback of the dataset is the limited number of NUTS-3 units with background stations (at best about half of the 402 NUTS-3 units), and the consequent need for gap filling using reanalysis data. Despite the refined scaling strategy used, this approach introduces a methodological discontinuity, whose implications must be investigated in, e.g., observational studies.

## Ethics Statement

The authors have read and follow the ethical requirements for publication in Data in Brief, confirming that the work presented in this article does not involve human subjects, animal experiments, or any data collected from social media platforms.

## CRediT authorship contribution statement

**Alexandre Caseiro:** Conceptualization, Resources, Methodology, Software, Validation, Formal analysis, Investigation, Data curation, Writing – original draft, Visualization. **Erika von Schneidemesser:** Conceptualization, Resources, Writing – review & editing, Supervision, Project administration, Funding acquisition.

## Data Availability

ZenodoAPExpose_DE (Original data)
